# Population prevalence, attributable risk, and attributable risk percentage for high methylmalonic acid concentrations in the post-folic acid fortification period in the US

**DOI:** 10.1186/1743-7075-9-2

**Published:** 2012-01-11

**Authors:** Vijay Ganji, Mohammad R Kafai

**Affiliations:** 1 Division of Nutrition, Byrdine F. Lewis School of Nursing and Health Professions, Georgia State University, Atlanta, GA 30302, USA; 2 Department of Mathematics, San Francisco State University, San Francisco, CA 94132, USA

**Keywords:** Age, methylmalonic acid, NHANES, population attributable risk, population attributable risk percentage

## Abstract

**Background:**

Serum methylmalonic acid (MMA) is regarded as a sensitive marker of vitamin B-12 status. Elevated circulating MMA is linked to neurological abnormalities. Contribution of age, supplement use, kidney dysfunction, and vitamin B-12 deficiency to high serum MMA in post-folic acid fortification period is unknown.

**Methods:**

We investigated prevalence, population attributable risk (PAR), and PAR% for high MMA concentrations in the US. Data from 3 cross-sectional National Health and Nutrition Examination Surveys conducted in post-folic acid fortification period were used (*n *= 18569).

**Results:**

Likelihood of having high serum MMA for white relative to black was 2.5 (*P *< 0.0001), ≥ 60 y old persons relative to < 60 y old persons was 4.0 (*P *< 0.0001), non-supplement users relative to supplement users was 1.8 (*P *< 0.0001), persons with serum creatinine ≥ 130 μmol/L relative to those with < 130 μmol/L was 12.6 (*P *< 0.0001), and persons with serum vitamin B-12 < 148 pmol/L relative to those with ≥ 148 pmol/L was 13.5 (*P *< 0.0001). PAR% for high MMA for old age, vitamin B-12 deficiency, kidney dysfunction, and non-supplement use were 40.5, 16.2, 13.3, and 11.8, respectively. By improving serum vitamin B-12 (≥ 148 pmol/L), prevalence of high MMA would be reduced by 16-18% regardless of kidney dysfunction.

**Conclusions:**

Old age is the strongest determinant of PAR for high MMA. About 5 cases of high serum MMA/1000 people would be reduced if vitamin B-12 deficiency (< 148 pmol/L) is eliminated. Large portion of high MMA cases are not attributable to serum vitamin B-12. Thus, caution should be used in attributing high serum MMA to vitamin B-12 deficiency.

## Introduction

Elevated circulating methylmalonic acid (MMA) is an emerging potential risk factor for neurodegenerative diseases and thus may be neurotoxic [1,2]. MMA interferes with energy production in mitochondria by inhibiting electron transport complex II [3,4]. Epidemiological studies have linked high circulating MMA with declined cognitive function [5,6]. Doubling serum MMA concentration from 0.25 to 0.50 μmol//L was associated with > 50% more rapid cognitive decline in a longitudinal study conducted in the UK [5]. In another longitudinal study in the US, serum MMA concentrations were predictive of rapid cognitive decline in older subjects [6].

Serum MMA is considered as a sensitive marker of tissue vitamin B-12 deficiency [7,8]. Although serum vitamin B-12 is widely used as a marker of vitamin B-12 deficiency [9], serum vitamin B-12 may not always reflect true vitamin B-12 status because some individuals with low-normal vitamin B-12 exhibit tissue vitamin B-12 deficiency [10]. In vitamin B-12 deficiency, serum MMA is derived from L-methylmalonyl CoA due to impaired function of methylmalonyl CoA mutase [11]. Adenosylcobalamin, a coenzyme of vitamin B-12, is required for the function of methylmalonyl CoA mutase, which converts methylmalonyl CoA to succinyl CoA [12].

Beginning January 1 1998, the FDA mandated that all processed cereals be fortified with folic acid in order to reduce the risk of neural tube defects (NTD) in newborn [13,14]. As a result, NTDs are reduced by 19% [15,16] owing to improved folate status [17-20]. Secondary to reduction in NTD, folic acid fortification has lowered circulating total homocysteine (tHcy) [17,18,21] and prevalence of anemia [22]. There are some concerns regarding possible negative impact of high folate status following folic acid fortification in those with vitamin B-12 deficiency [23-25]. These concerns stem from reports suggesting that high folic acid intake may lead to the correction of hematological abnormalities associated with vitamin B-12 deficiency [26,27] which may lead to delay in diagnosis of vitamin B-12 deficiency leading to irreversible neuropathy [28,29]. Although the precise mechanism through which high folate status causes harm in those with vitamin B-12 deficiency is not known, recently, Selhub et al [30] very elegantly proposed how high serum MMA associated with low vitamin B-12 and high folate status disrupts vitamin B-12 homeostasis.

Vitamin B-12 status and kidney function are two important determinants of MMA [31-33]. Prevalence of high serum MMA in older Americans was 20% in the pre-folic acid fortification period [31]. In this study, we used nationally representative surveys to yield large sample size. Because serum MMA is regarded as a sensitive marker of vitamin B-12 deficiency and is elevated in kidney dysfunction, a common condition in older persons and that elevated MMA is related to negative health outcomes [1-6], it is important to know the contribution of vitamin B-12 deficiency, kidney dysfunction, and older age to the burden of circulating high MMA. Moreover, there are very limited data on serum MMA and its relation with race-ethnicity [34]. Therefore, the objective of this study was to investigate population prevalence estimates, population attributable risk (PAR), and PAR% for high serum MMA in the post-folic acid fortification period for US population.

## Methods and Subjects

### Description of survey and study sample

National Health and Nutrition and Examination Surveys (NHANES) are based on a complex, stratified, multistage probability sample survey conducted by National Center for Health Statistics (NCHS). Demographic, socioeconomic, dietary, and health related data were collected in the participants' home as part of the household interview. A physician administered examination component is part of the assessment done on household interviewed persons in Mobile Examination Centers (MEC). Rest of the examination assessment on individuals is performed by health technicians in MEC. In NHANES, young children, older persons, non-Hispanic black, and Mexican American/Hispanic were oversampled to yield reliable estimates for these groups.

Beginning 1999, NHANESs were conducted as continuous, annual surveys rather than the periodic surveys. In this study, NHANES 1999-2000 [35], NHANES 2001-2002 [36], and NHANES 2003-2004 [37] were concatenated into one analytic database, NHANES 1999-2004, as per guidelines from the NCHS. The description of these surveys has been reported in detail elsewhere [38-40]. Briefly, NHANES 1999-2000 was conducted between March 1999 and December 2000 on 9965 individuals (all were home interviewed; 9282 were examined in MECs); NHANES 2001-2002 was conducted between January 2001 and December 2002 on 11039 individuals (all were home interviewed; 10477 were examined in MECs); and NHANES 2003-2004 was conducted between January 2003 and December 2004 on 12761 individuals (10122 were home interviewed; 9643 were examined in MECs).

The combined 3 cycles of NHANES 1999-2004 yielded a sample size of 31126. In data analysis, individuals who reported their race-ethnicity as "Other" were excluded *(n *= 884). This "Other" category represented people with mixed races and individuals belonging to race-ethnicities other than non-Hispanic white, non-Hispanic black, or Mexican American/Hispanic. There were no missing data for sex, age, and race-ethnicity. Individuals with missing data for supplement use (*n *= 28), serum creatinine (*n *= 4093), serum vitamin B-12 (*n *= 18), and serum MMA (*n *= 7534) were excluded. After applying the aforementioned exclusion criteria, the final study sample consisted of 18569 subjects. Sample size for persons with normal kidney function was 18238.

### Measurements

Depending on the age of the participant, data were collected on body measurements, demography, physical function, health condition, lifestyle behaviors, biochemical measurements of blood and urine, and diet intake. Because we used serum creatinine as one of the variables related to serum MMA and in NHANES serum creatinine concentrations were measured only in persons > 12 y old age, this current study was based on the sample > 12 y old age group. Other variables such as supplement use, serum vitamin B-12, and serum MMA had no impact on the age of this study sample. Blood was collected from venipuncture in the MECs according to the standard protocols. Individuals who took vitamin/mineral supplements 1 month prior to the survey were categorized as supplement users. Serum MMA was measured using gas chromatography/mass spectrophotometry. Serum vitamin B-12 was measured using Quantaphase II radioassay kit (Biorad Laboratories, Hercules, CA). Serum creatinine was measured using an autoanalyzer (Hitachi Model 917 for NHANES 1999-2002; Beckman Synchron LX20 for NHANES 2003-2004). Sex, race-ethnicity, age, supplement use, serum creatinine, and serum vitamin B-12 were selected as important variables that are associated with serum MMA concentrations. Detailed methodology on laboratory procedures are described elsewhere [41-43].

Serum concentration of > 350 nmol/L was used to define high MMA [44]. Serum creatinine concentrations ≥ 130 μmol/L was used to indicate impaired kidney function [45]. Vitamin B-12 deficiency was defined as having serum vitamin B-12 concentrations < 148 pmol/L [30,31,46].

### Statistical analysis

SUDAAN (SAS-callable) statistical software (SUDAAN for Windows, version 10.0, Research Triangle Institute, Research Triangle Park, NC) was used to account for complex survey design. In data analysis, to account for differential probabilities of selection and adjustments for non-coverage and non-response bias, sample weights, primary sampling units, and stratification variables were included as per NCHS's guidelines. Also, SAS (SAS for Windows, version 9.1, SAS Institute Inc, Cary, NC) was used in conjunction with SUDAAN to manage and analyze data files.

Because of non-normality distribution of serum MMA concentrations, natural logarithmic transformation was used to satisfy the requirement for normality. Standard errors were generated with Taylor Linearization Series as this method takes the complex NHANES design and sampling in to account. Odds ratio (OR) and 95% confidence intervals (CI) were determined for serum MMA > 350 nmol/L with multivariate logistic regression analysis. Statistical significance was set at *P *< 0.05.

PAR (prevalence of a condition/disease in the population due to the presence of risk factor or prevalence of a condition/disease in the population that would be reduced if risk factor was removed) and PAR% (percent of prevalence of a condition/disease in the population due to presence of risk factor or percent of prevalence of a condition/disease in the population that would be reduced if risk factor was removed) for serum MMA > 350 nmol/L were calculated for non-Hispanic white relative to combined non-Hispanic black and Mexican American/Hispanic (non-white), older persons (≥ 60 y) relative to younger persons (< 60 y), non-supplement users relative to supplement users, persons with serum creatinine ≥ 130 μmol/L relative to serum creatinine < 130 μmol/L, and persons with serum vitamin B-12 < 148 pmol/L relative to serum vitamin B-12 ≥ 148 pmol/L. Non-Hispanic white, older persons (≥ 60 y), nonsupplement users, persons with serum creatinine ≥ 130 μmol/L, and persons with serum vitamin B-12 < 148 pmol/L were used as persons at risk for high serum MMA concentrations. Their counterparts, non-non-Hispanic white (non-white), younger persons (< 60 y), supplements users, persons with serum creatinine < 130 μmol/L, and persons with serum vitamin B-12 ≥ 148 pmol/L were used as referent groups. In order to create a dichotomous variable for race-ethnicity, persons of non-Hispanic black and Mexican American/Hispanic were combined into one category, non-non-Hispanic white (non-white). This enabled us to compare white with non-white population. Age variable was divided into < 60 y and ≥ 60 y old categories to create a dichotomized variable. We chose this age cut off because the serum MMA concentrations started in increase more around the age of 60 y based on our initial observation of serum MMA in this study sample. PAR and PAR% were not calculated for sex because this variable was not significantly related to serum MMA in the logistic regression (*P *= 0.98). All PAR and PAR% calculations were based on weighted sample. Because in kidney dysfunction, MMA concentrations are elevated, PAR and PAR% also calculated after excluding persons with serum creatinine ≥ 130 μmol/L. This allowed us to assess the impact of kidney dysfunction on PAR and PAR% for serum MMA. Formulas for calculation of PAR and PAR% for high serum MMA are given below [47,48].

$$\text{PAR}=\;\left(\text{Prevalence of high MM}{\text{A}}_{\text{Total}\;\text{sample}}\;\times \text{ 1}00\right)-\left(\text{Prevalence of high MM}{\text{A}}_{\text{Referent group}}\;\times 100\right)$$

$$\text{PAR\% }=\;\frac{\text{Prevalence of high MM}{\text{A}}_{\text{Total}\;\text{sample}}-\text{Prevalence of high MM}{\text{A}}_{\text{Referent group}}}{\text{Prevalence of high MM}{\text{A}}_{\text{Total}\;\text{sample}}}\times 100$$

$$\text{Prevalence of high MM}{\text{A}}_{\text{Total }\;\text{sample}}=\text{Cases}\;\text{of}\;\text{high MM}{\text{A}}_{\text{Total }\;\text{sample}}/)\text{Sample}\;\text{siz}{\text{e}}_{\text{Total}}$$

$$\text{Prevalence of high MM}{\text{A}}_{\text{Referent group}}=\text{Cases}\;\text{of}\;\text{high MM}{\text{A}}_{{}_{\text{Referent group}}}/)\text{Sample}\;\text{siz}{\text{e}}_{\text{Referent group}}$$

## Results

After excluding missing values, 18569 persons had measured serum MMA concentrations in NAHNES 1999-2004. Men were ≈49% (*n *= 9020), women were ≈51% (*n *= 9549), non-Hispanic white were ≈44% (*n *= 8170), non-Hispanic black were ≈23% (*n *= 4351), Mexican American/Hispanic were ≈33% (*n *= 6048), adolescents (12 - < 18 y) were ≈24% (*n *= 4546), and older persons (≥ 60 y) were ≈24% (*n *= 4427). About 41% of the study participants reported that they consumed supplements during 1 month prior to the survey (Table 1**)**.

**Table 1:** Subject characteristics of study population: National Health and Nutrition Examination Surveys (NHANES), 1999-2004^1^

Characteristic	Value
All subjects, *n*	18569
Men, *n*(%)	9020 (49)
Women, *n *(%)	9549 (51)
Race-ethnicity	
Non-Hispanic white, *n *(%)	8170 (44)
Non-Hispanic black, *n *(%)	4351 (23.4)
Mexican American/Hispanic, *n *(%)	6048 (32.6)
Age	
12- < 18 y, *n (%)*	4546 (24)
≥ 60 y, *n (%) *	4427 (23.8)
Supplement users, *n *(%)	7681 (41)
Non-supplement users^2^, *n *(%)	10888 (59)
Serum creatinine ≥ 130 μmol/L, *n *(%)	331 (1.8)
Serum vitamin B-12 < 148 pmol/L, *n *(%)	349 (1.9)
Methylmalonic acid (nmol/L)^3^	129.3 (99.4, 169.4)
Prevalence of high methylmalonic acid	
All subjects (%)^4^	3.1 ± 0.21
Serum creatinine < 130 μmol/L (%)^4^	2.7 ± 0.2

^1^NHANES 1999-2000, 2001-2002, and 2003-2004 were combined into one analytic data set, 1999-2004. NHANESs 1999-2004 were conducted in the post-folic acid fortification period.

^2^Persons who did not take vitamin/mineral supplements 1 month prior to the survey

^3^Median and 25^th ^and 75^th ^percentiles. Distribution of serum MMA was not normal

^4^Methylmalonic acid concentration > 350 nmol/L. Prevalence mean ± SE

Prevalence (%) and *OR*s (95% CI) for high serum MMA by sex, race-ethnicity, age, supplement use, serum creatinine, and serum vitamin B-12 for US population in the post-folic acid fortification period are presented in Table 2. Overall prevalence of high serum MMA was 3.1%. Odds of having high serum MMA was significantly higher in non-Hispanic white (prevalence, 3.4%; *OR*, 2.48; *P *< 0.001) and Mexican American/Hispanic (prevalence, 3.0%; *OR*, 2.77; *P *< 0.0003) relative to non-Hispanic black (prevalence, 0.2%), significantly higher in persons ≥ 60 y old (prevalence, 8.3%; *OR*, 4.0; *P *< 0.0001) relative to persons < 60 y old (prevalence, 1.9%), significantly higher in nonsupplement users (prevalence, 3.5%; *OR*, 1.82; *P *< 0.0001) relative to supplement users (prevalence, 2.8%), significantly higher in persons with serum creatinine ≥ 130 μmol/L (prevalence, 34.1%; *OR*, 12.6; *P *< 0.0001) relative to persons with serum creatinine < 130 μmol/L (prevalence, 2.7%), and significantly higher in persons with serum vitamin B-12 < 148 pmol/L (prevalence, 27.6%; *OR*, 13.5; *P *< 0.0001) relative to persons with serum vitamin B-12 ≥ 148 pmol/L (prevalence, 2.6%). Sex was not significantly related to high MMA concentrations (*P *= 0.98).

**Table 2:** Prevalence and likelihood of having high methylmalonic acid (MMA) in the post-folic acid fortification period: National Health and Nutrition Examination Surveys (NHANES), 1999-2004^1^

Characteristic	Prevalence^2^*%*	*OR *(95% *CI*)^3^	*P*-Value^4^	*P*-Value^5^
Sex				0.98
Men (*n *= 9020)	3.2	1.0 (0.83, 1.19)	NS	
Women (*n *= 9549)^6^	3.0	1.0	--	
Race-ethnicity				< 0.0001
Non-Hispanic white (*n *= 8170)	3.4	2.48 (1.78, 3.45)	< 0.001	
Non-Hispanic black (*n *= 4351)^6^	0.2	1.0	--	
Mexican American/Hispanic (*n *= 6048)	3.0	2.77 (1.86, 4.14)	0.0003	
Age				< 0.0001
< 60 y (*n *= 14142)^6^	1.9	1.0	--	
60 y (*n *= 4427)	8.3	4.01 (3.26, 4.92)	< 0.0001	
Supplement use^7^				< 0.0001
Yes (*n *= 7681)^6^	2.8	1.0	--	
No (*n *= 10888)	3.5	1.82 (1.40, 2.37)	< 0.0001	
Serum creatinine^8^				< 0.0001
< 130 μmol/L (*n *= 18238)^6^	2.7	1.0	--	
130 μmol/L (*n *= 331)	34.1	12.6 (8.99, 17.7)	< 0.0001	
Serum vitamin B-12^9^				< 0.0001
< 148 pmol/L (*n *= 349)	27.6	13.5 (9.29, 19.6)	< 0.0001	
148 pmol/L (*n *= 18220)^6^	2.6	1.0	--	

^1^*n *= 18569. NHANES 1999-2000, 2001-2002, and 2003-2004 were combined into one analytic data set, 1999-2004. NHANESs 1999-2004 were conducted in the post-folic acid fortification period. Overall prevalence of serum MMA > 350 nmol/L was 3.1 ± 0.2 (% and SE)

^2^Population prevalence of serum MMA > 350 pmol/L was based on weighted sample size

^3^Odds ratio and 95% Wald confidence intervals in the multivariate logistic regression analysis

^4^Significance in comparison to the referent category within the variable (*P *for Wald χ^2^)

^5^Overall significance of variable in the logistic regression model (*P *for Wald χ^2^)

^6^Referent group

^7^Persons who took vitamin/mineral supplements 1 month prior to the survey

^8^Impaired renal function is defined as having serum creatinine ≥ 130 μmol/L

^9^Vitamin B-12 deficiency is defined as having serum vitamin B-12 < 148 pmol/L

After excluding persons with high serum creatinine (≥ 130 μmol/L), prevalence (%) and *OR*s (95% CI) for high serum MMA by race-ethnicity, age, supplement use, and serum vitamin B-12 for US population in the post-folic acid fortification period are presented in Table 3. Overall, prevalence of high serum MMA in those with normal renal function was 2.7%. In this population, age, race-ethnicity, supplement use, and serum vitamin B-12 were significantly related to serum high MMA. Likelihood of having high serum MMA was significantly lower in non-Hispanic black relative to non-Hispanic white (0.8% vs. 2.8%; *OR*, 0.28; *P *< 0.0001), significantly higher in ≥ 60 y old persons relative to < 60 y old persons (6.8% vs. 1.8%; *OR*, 4.38; *P *< 0.0001), significantly higher in nonsupplement users relative to supplement users (3.1% vs. 2.3%; *OR*, 1.93; *P *< 0.0001), and significantly higher in those with vitamin B-12 < 148 pmol/L compared to those with vitamin B-12 ≥ 148 pmol/L (26.3% vs. 2.2%; *OR*, 13.3, *P *< 0.0001).

**Table 3:** Prevalence and likelihood of having high serum methylmalonic acid (MMA) in persons with serum creatinine < 130 μmol/L concentrations in the post-folic acid fortification period: National Health and Nutrition Examination Surveys (NHANES), 1999-20041

Characteristic	Prevalence^2^ *%*	*OR *(95% *CI*)^3^	*P*-Value^4^	*P*-Value^5^
Race-ethnicity				< 0.0001
Non-Hispanic white (*n *= 7995)^6^	3.0	1.0	--	
				
Non-Hispanic black (*n *= 4247)	0.8	0.28 (0.18, 0.44)	< 0.0001	
				
Mexican American/Hispanic (*n *= 5996)	2.8	1.1 (0.81, 1.50)n	n/s	
Age				< 0.0001
< 60 y (*n *= 14090)^6^	1.8	1.0	--	
60 y (*n *= 4148)	6.8	4.38 (3.58, 5.36)	< 0.0001	
Supplement use^7^				< 0.0001
Yes (*n *= 7506)^6^	2.3	1.0	--	
No (*n *= 10732)	3.1	1.93 (1.48, 2.52)	< 0.0001	
Serum vitamin B-12^8^				< 0.0001
< 148 pmol/L (*n *= 343)	26.3	13.3 (9.0, 19.5)	< 0.0001	
148 pmol/L (*n *= 17895)^6^	2.2	1.0	--	

^1 ^n = 18238; NHANES 1999-2000, 2001-2002, and 2003-2004 were combined into one analytic data set, 1999-2004. NHANESs 1999-2004 were conducted in the post-folic acid fortification period. Data associated with sex variable are not shown because in logistic regression sex was not significantly related to serum MMA concentrations (*P *< 0.98).

^2^Serum MMA > 350 pmol/L

^3^Odds ratio and 95% Wald confidence intervals in the multivariate logistic regression analysis

^4^Significance in comparison to the referent category within the variable (*P *for Wald χ^2^)

^5^Overall significance of variable (sex, race-ethnicity, supplement use, age, serum creatinine, or serum vitamin B-12) in the logistic regression model (*P *for Wald χ^2^)

^6^Referent group

^7^Persons who took vitamin/mineral supplements 1 month prior to the survey

^8^Vitamin B-12 deficiency is defined as having serum vitamin B-12 < 148 pmol/L

PAR and PAR% for high serum MMA by race-ethnicity, age, supplement use, serum creatinine, and serum vitamin B-12 for US population in the post-folic acid fortification period are presented in Table 4. PARs for serum MMA > 350 nmol/L were ≈8 cases/1000 people (0.774) for non-Hispanic white relative to non-non-Hispanic white, ≈13 cases/1000 people (1.262) for older persons (≥ 60 y) relative to younger persons (< 60 y), ≈4 cases/1000 people (0.368) for non-supplement users relative to supplement users, ≈4 cases/1000 people (0.415) for serum creatinine ≥ 130 μmol/L relative to serum creatinine < 130 μmol/L, and ≈5 cases/1000 people (0.506) for serum vitamin B-12 < 148 pmol/L relative to serum vitamin B-12 ≥ 148 pmol/L. PAR%s for serum MMA > 350 nmol/L were ≈25% for non-Hispanic white in relation to non-non-Hispanic white, ≈41% for older persons (≥ 60 y) in relation to younger persons (< 60 y), ≈12% for nonsupplement users in relation to supplement users, ≈13% for serum creatinine ≥ 130 μmol/L in relation to serum creatinine < 130 μmol/L, and ≈16% for serum vitamin B-12 < 148 pmol/L in relation to serum vitamin B-12 ≥ 148 pmol/L.

**Table 4:** Population attributable risk (PAR) and population attributable risk percentage (PAR%) for high serum methylmalonic acid (MMA) in the National Health and Nutrition Examination Surveys (NHANES), 1999-2004^1^

Characteristic	Cases^2^	PAR^3^	PAR%^4^
Race-ethnicity^5^			
Non-Hispanic white (*n *= 8170)	356	0.774	24.8
Non non-Hispanic white (*n *= 10399)^6, 7^	230	--	--
Age^5^			
< 60 y (*n *= 14142)^6^	204	--	--
60 y (*n *= 4427)	382	1.262	40.5
Supplement use^8, 9^			
Yes (*n *= 7681)^6^	252	--	--
No (*n *= 10888)	334	0.368	11.8
Serum creatinine^8, 10^			
< 130 μmol/L (*n *= 18238)^6^	477	--	--
130 μmol/L (*n *= 331)	109	0.415	13.3
Serum vitamin B-12^8, 11^			
< 148 pmol/L (*n *= 349)	111	0.506	16.2
148 pmol/L (*n *= 18220)^6^	475	--	--

^1 ^n = 18569; NHANES 1999-2000, 2001-2002, and 2003-2004 were combined into one analytic data set, 1999-2004. NHANESs, 1999-2004 were conducted after the folic acid fortification commenced. PAR and PAR% for sex were not presented because sex variable was not significantly related to high serum MMA in the logistic regression model (*P *= 0.98).

^2^Number of cases with serum MMA > 350 nmol/L

^3^Prevalence of a condition/disease in the population due to the presence of risk factor or prevalence of a condition/disease in the population that would be reduced if risk factor was removed. PAR = (Prevalence of high MMA_Total sample _× 100) - (Prevalence of high serum MMA_Referent group _× 100). Prevalence of high serum MMA_Total sample _= Cases of high serum MMA_Total sample_/Sample size_Total_. Prevalence of high serum MMA_Referent group _= Cases of high serum MMA_Referent group_/Sample size_Referent group_. PAR and PAR% were calculated based on weighted sample. Weighted sample was used to account for differential probabilities of selection and adjustments for non-coverage and non-response bias.

^4^Percent of prevalence of a condition/disease in the population due to presence of risk factor or percent of prevalence of a condition/disease in the population that would be reduced if risk factor was removed. PAR% was calculated based on weighted sample size. PAR% = (Prevalence of high serum MMA_Total sample _- Prevalence of high serum MMA_Referent group _÷ Prevalence of high serum MMA_Total sample_) x100

^5^Non-modifiable risk factor for high serum MMA

^6^Referent group

^7^In order to achieve a dichotomous variable for race-ethnicity, non-Hispanic black and Mexican American/Hispanic were combined into one category, non-non-Hispanic white (non-white). Cases of serum MMA > 350 nmol/L for non-Hispanic black and Mexican American/Hispanic were 65 and 165, respectively.

^8^Modifiable risk factor for high serum MMA

^9^Persons who took vitamin/mineral supplements 1 month prior to the survey

^10^Kidney dysfunction was defined as having serum creatinine ≥ 130 μmol/L

^11^Vitamin B-12 deficiency was defined as having serum vitamin B-12 < 148 pmol/L

Additionally, to evaluate the impact of kidney dysfunction on the relation of serum MMA with age, race-ethnicity, supplement use, and serum vitamin B-12, we calculated PAR and PAR% after excluding persons with high serum creatinine (≥ 130 μmol/L). In this subset population, PAR for serum high MMA were ≈8 cases/1000 people (0.78) for non-Hispanic white relative to combined non-Hispanic black and Mexican/Hispanic, ≈10 cases/1000 people (0.95) for ≥ 60 y old persons relative to < 60 y old persons, ≈4 cases/1000 people (0.44) for nonsuplement users relative to supplement users, and ≈5 cases/1000 people (0.485) for serum vitamin B-12 < 148 pmol/L group relative to vitamin B-12 ≥ 148 pmol/L group (Table 5).

**Table 5:** Population attributable risk (PAR) and population attributable risk percentages (PAR%) for high serum methylmalonic acid (MMA) for persons with serum creatinine < 130 μmol/L in the National Health and Nutrition Examination Surveys (NHANES), 1999-2004^1^

Characteristic	Cases^2^	PAR^3^	PAR%^4^
Race-ethnicity^5^			
Non-Hispanic white (*n *= 7995)	297	0.783	29.0
Non non-Hispanic white (*n *= 10243)^6, 7^	180	--	--
Age^5^			
< 60 y (*n *= 14090)^6^	188	--	--
60 y (*n *= 4148)	289	0.950	35.2
Supplement use^8, 9^			
Yes (*n *= 7506)^6^	192	--	--
No (*n *= 10732)	285	0.444	16.4
Serum vitamin B-12^8, 10^			
< 148 pmol/L (*n *= 343)	105	0.485	18.0
148 pmol/L (*n *= 17895)^6^	372	--	--

^1^*n *= 18238; NHANES 1999-2000, 2001-2002, and 2003-2004 were combined into one analytic data set, 1999-2004. NHANESs, 1999-2004 were conducted after the folic acid fortification commenced. PAR and PAR% for sex were not presented because sex variable was not significantly related to high serum MMA in the logistic regression model (*P *= 0.98)

^2^Number of cases with serum MMA > 350 nmol/L

^3^Incidence of a condition/disease in the population due to the presence of risk factor or incidence of a condition/disease in the population that would be reduced if risk factor was removed. PAR was calculated based on weighted sample size. PAR = (Incidence of high MMA_Total sample _× 100) - (Incidence of high serum MMA_Referent group _× 100). Incidence of high serum MMA_Total sample _= Cases of high serum MMA_Total sample_/Sample size_Total_. Incidence of high serum MMA_Referent group _= Cases of high serum MMA_Referent group_/Sample size_Referent group_.

^4^Percent of incidence of a condition/disease in the population due to presence of risk factor or percent of incidence of a condition/disease in the population that would be reduced if risk factor was removed. PAR% was calculated based on weighted sample size. PAR% = (Incidence of high serum MMA_Total sample _- Incidence of high serum MMA_Referent group _÷ Incidence of high serum MMA_Total sample_) x100

^5^Non-modifiable risk factor for high serum MMA

^6^Referent group

^7^In order to achieve a dichotomous variable for race-ethnicity, non-Hispanic black and Mexican American/Hispanic were combined into one category, non-non-Hispanic white.

^8^Modifiable risk factor for high serum MMA

^9^Persons who took vitamin/mineral supplements 1 month prior to the survey

^10^Vitamin B-12 deficiency was defined as having serum vitamin B-12 < 148 pmol/L

## Discussion

In this report, we present the first data on prevalences, PAR, and PAR% for high serum MMA in the post-folic acid fortification period utilizing the data from surveys of US population. The strength of this study is that we combined 3 NHANES cycles into one database, which not only resulted in a large sample size (*n *= 18569) but also increased the precision of MMA estimate. We found that likelihood of having high serum MMA was significantly higher in white, older persons, nonsupplement users, and persons with high serum creatinine and low vitamin B-12 compared to their counterparts. Old age, vitamin B-12 deficiency, renal dysfunction, and non-supplement use contributed to PAR and PAR% for high serum MMA.

In vitamin B-12 deficiency not only MMA but also other undesirable products such as propionyl CoA, methyl citrate (formed from condensation of propionyl CoA and acetyl CoA by citrate synthase), and tHcy are accumulated. Rare genetic defects in methlymalonyl CoA mutase (*mut *0 and *mut*-) and synthesis of defective adenosylcobalamin are sources of MMA in some individuals [49]. Other sources of MMA include odd chain fatty acids, certain amino acids, and intestinal bacteria [10,50]. However, there were no data to what extent these sources contribute to serum MMA. The deleterious effects of vitamin B-12 deficiency on nervous system are attributed to reduced methylation reactions due to diminished supply of S-adenosylmethionine and also possibly due to elevated MMA, propionyl CoA, methylcitrate [1-4], and tHcy [2,51].

In this study, the overall prevalence of serum MMA > 350 pmol/L is 3.1%. In older adults (≥ 60 y), it is 8.3%. Prevalence of serum vitamin B-12 < 148 pmol/L is 2.0%. In the prefortification period, prevalence of high MMA (> 370 nmol/L) in persons aged ≥ 65 y was 16-18% (*n *= 1145) [31]. In the Los Angeles elderly people (> 60 y, *n *= 725), 16.6% had high MMA (> 370-376 pmol/L) and 11.8% had serum vitamin B-12 < 140 pmol/L [32]. In the Framingham study, 8% adults (26-83 y) had plasma vitamin B-12 < 148 pmol/L [52]. In the Framingham elderly population (67-96 y), 40.5% (222/548) had serum vitamin B-12 < 258 pmol/L [9]. These studies were based on white population. Thus, the existing data on high MMA and low vitamin B-12 in the US vary by population studied and criteria used to define vitamin B-12 deficiency.

In this study, persons in the ≥ 60 y old age group are > 4 times likely to have high serum MMA compared to those in the younger age group (*P *< 0.001). A contributing factor for high serum MMA in older persons is kidney dysfunction. In a separate analysis on ≥ 60 y old population, the prevalence of high MMA in those with serum creatinine ≥ 130 μmol/L is ≈33% (93/279) compared to ≈7% (289/4148) in those with serum creatinine < 130 μmol/L. Thus, a large portion of high MMA cases can be attributed to renal dysfunction in older adults. Further, vitamin B-12 deficiency is more common in older persons primarily due to declined absorption of vitamin B-12 from gut due to decreased secretion of acid and pepsin from gastric atrophy which is a result of bacterial colonization in the stomach [53,54]. Acid and pepsin are needed to release the protein- bound vitamin B-12 prior to its absorption. In some older adults, use of medications such as H_2 _receptor antagonists and proton pump inhibitors also lead to low vitamin B-12 status due to malabsorption of protein-bound vitamin B-12 from achlorhydria [55]. In some, poor vitamin B-12 status is attributed to autoimmune disorder associated with *H. pylori *infection [56]. All these lead to elevated MMA.

Age is the strongest determinant of PAR (1.26) and PAR% (40.5) for high serum MMA. After excluding persons with kidney dysfunction, age still greatly contributed to PAR (0.95) and PAR% (35.2) for high MMA. Thus, kidney dysfunction contributed ≈3 (1.26-0.95)/1000 to the prevalence of serum high MMA. PAR and PAR% for high serum MMA for serum vitamin B-12 < 148 pmol/L were ≈0.51 and 16.2, respectively. After excluding persons with kidney dysfunction, the PAR and PAR% for high MMA for low serum vitamin B-12 were ≈0.49 and 18.0, respectively (Table 4). This suggests that low serum vitamin B-12 (< 148 pmol/L) is not as strong determinant of PAR for high serum MMA.

In this study, PAR% for high MMA associated with non-Hispanic white race is ≈25%, which suggests that the race-ethnicity is a major determinant of serum MMA in the US. In a separate analysis on this sample (data not shown), we observed that non-Hispanic white had significantly higher prevalence of serum vitamin B-12 < 148 pmol/L (2.6%) compared to non-Hispanic black (0.9%) or Mexican American/Hispanic (1.6%). Thus, it is not surprising that non-Hispanic white had higher prevalence of serum MMA compared to non-Hispanic black or Mexican American/Hispanic. These results support the observations made by others previously [32,33]. In the elderly, Morris et al [31] reported high MMA in non-Hispanic white but the prevalence in those subjects was similar to non-Hispanic black. Causes for differences between races regarding MMA metabolism needs further attention.

To date, there is no consensus regarding the cutoff values for serum vitamin B-12 to define vitamin B-12 deficiency due to lack of diagnostic specificity and sensitivity [57]. In few cases, serum vitamin B-12 does not reflect true vitamin B-12 status because only 20-30% is bound to holotranscobalamin (holoTC), a biologically active vitamin B-12 fraction, capable of delivering vitamin B-12 to tissues and the remaining majority is bound to haptocorrin [58]. Because serum vitamin B-12 assay does not distinguish between haptocrrin and holoTC

fractions, measurement of holoTC has been suggested as a sensitive and early marker of vitamin B-12 deficiency [59]. However, like MMA, holoTC is also elevated in renal dysfunction [60] leaving no universal gold standard for the measurement of vitamin B-12 deficiency.

There are some assumptions in calculating PAR and PAR%. These are (a) cause and effect relationship exists between serum MMA and renal dysfunction and serum vitamin B-12 and (b) serum creatinine > 130 μmol/L, serum vitamin B-12 < 148 pmol/L, and serum MMA > 350 nmol/L were used to define kidney dysfunction, vitamin B-12 deficiency, and high serum MMA, respectively.

## Conclusions

In conclusion, a reduction of ≈5 cases of high MMA/1000 people is expected if serum vitamin B-12 in the US is improved to or > 148 pmol/L. Such a decrease represents ≈16% reduction of prevalence of high MMA. After excluding persons with kidney dysfunction, PAR% for high MMA for vitamin B-12 deficiency is marginally increased (from 16.2 to 18) which further strengthens our notion that even in the absence of renal dysfunction a large portion of high MMA cases might not be affected with the improvement in vitamin B-12 status. Regardless of kidney function, prevalence of high serum MMA would be reduced by 16-18% (PAR is ≈5 cases/1000 people) in the US population if the risk factor, vitamin B-12 deficiency is eliminated.

Because a large portion of high serum MMA cases in the US are not related to vitamin B-12 status (serum vitamin B-12 < 148 pmol/L), caution should be used in attributing high serum MMA to vitamin B-12 deficiency even in the absence of kidney dysfunction. Given the depressed absorption of vitamin B-12 and increased prevalence of renal dysfunction, it is unlikely that increased oral intake of vitamin B-12 would dramatically reduce the prevalence of high serum MMA in older adults. It is important to monitor the indicators of vitamin B-12 status such as serum MMA concentrations, on a continuous basis because of possible negative impact of high folic acid intakes on vitamin B-12 homeostasis in the post-folic acid fortification period.

## Competing interests

The authors declare that they have no competing interests.

## Authors' contributions

VG contributed to the study design and writing of the manuscript. MRK contributed to data acquisition, data management, and data analysis. Both authors contributed to the interpretation of results and review, revision, and editing of the manuscript. All authors read and approved the final version of the manuscript.
